# Validating Healthy Eating Index, Glycemic Index, and Glycemic Load with Modern Diets for E-Health Era

**DOI:** 10.3390/nu15051263

**Published:** 2023-03-03

**Authors:** Zhao-Feng Chen, Joyce D. Kusuma, Shyang-Yun Pamela K. Shiao

**Affiliations:** 1 Chung-Ho Memorial Hospital, Kaohsiung Medical University, Kaohsiung 80756, Taiwan; 2 The Villages Health, The Villages, FL 32162, USA; 3 Center for Biotechnology and Genomic Medicine, Medical College of Georgia, Augusta University, Augusta, GA 30912, USA

**Keywords:** healthy eating, glycemic index, glycemic load, modern diets, artificial intelligence, validated predictive modeling

## Abstract

Predictors of healthy eating parameters, including the Healthy Eating Index (HEI), Glycemic Index (GI), and Glycemic Load (GL), were examined using various modern diets (n = 131) in preparation for personalized nutrition in the e-health era. Using Nutrition Data Systems for Research computerized software and artificial intelligence machine-learning-based predictive validation analyses, we included domains of HEI, caloric source, and various diets as the potentially modifiable factors. HEI predictors included whole fruits and whole grains, and empty calories. Carbohydrates were the common predictor for both GI and GL, with total fruits and Mexican diets being additional predictors for GI. The median amount of carbohydrates to reach an acceptable GL < 20 was predicted as 33.95 g per meal (median: 3.59 meals daily) with a regression coefficient of 37.33 across all daily diets. Diets with greater carbohydrates and more meals needed to reach acceptable GL < 20 included smoothies, convenient diets, and liquids. Mexican diets were the common predictor for GI and carbohydrates per meal to reach acceptable GL < 20; with smoothies (12.04), high-school (5.75), fast-food (4.48), Korean (4.30), Chinese (3.93), and liquid diets (3.71) presenting a higher median number of meals. These findings could be used to manage diets for various populations in the precision-based e-health era.

## 1. Introduction

Healthy eating of essential nutrients is vital for nutrigenomics pathways to prevent chronic diseases in vulnerable populations of various social–ethnic contexts presenting inflammatory risks in the e-health era [1,2,3,4,5]. Healthy eating includes sufficient intakes of fruits, vegetables, grains, dairy, proteins, nuts and oils; while limiting saturated fats, salt, and empty calories [6,7,8,9]. The convenience of processed and pre-packaged foods loaded with saturated fats, empty calories (sugar and alcohol), and sodium in modern societies could be limited to improve diet quality [10]. Thus, the Healthy Eating Index (HEI), Glycemic Index (GI), and Glycemic Load (GL) could be used to assess diet quality in modern times [11,12,13,14]; with higher HEI, lower GI, and lower GL scores being associated with decreased inflammation, cancer risk, and chronic diseases [3,15,16].

The amount of carbohydrates in the diet contributes to the GI and GL, which have been used to evaluate healthy diets, by their potential to promote inflammation and the risk of cancer [17,18]. In recent years, related professions, including the American Diabetes Association (ADA), as well as medical and health professionals, further suggested that adults with diabetes could aim to eat 45 to 60 g of carbohydrates with each meal and 15–20 g per snack to control for acceptable GL of <20 for more stable blood glucose levels [19,20,21,22,23,24]. The elements of HEI, including fruits, grains, vegetables, and empty calories, could be major sources of carbohydrates. However, scientific data connecting the amount of carbohydrates with the sources of food categories per meal to reach an ideal GL of <20 per meal is lacking. Additionally, while mobile technologies evolved with accessibility to assess dietary nutrient intakes per meal and food items, the validations on the accuracy of essential nutrients with meals are lacking, limited to only the caloric nutrients but not other essential nutrients [2,25]. Furthermore, current dietary assessments have been validated with daily dietary diaries and longer durations (Food Frequency Questionnaires: per week and longer duration, with the need of adjusting for fat contents), but not per meal [2,25]. Therefore, in preparation for personalized nutrition in e-health era, further validations with the efforts to identify modifiable factors for health eating parameters per meal and daily diets are necessary.

Healthy eating could prevent diseases for various ethnic populations, and the parameters of HEI could be used as modifiable factors for disease prevention [3,4,5]. Previously, using actual human diets across various ethnic groups, we validated the parameters of HEI, with predictors of total HEI score including whole fruits, milk, whole grains, saturated fats, oils and nuts (>80 as good score) [3]. Additionally, some parameters of HEI with the content of carbohydrates such as milk, empty calories, and vegetables were also predictors of GI scores (≤55 as good GI score included milk; and median GI score of <53.8 included milk, empty calories, and dark greens) in cancer patients having diabetes and chronic inflammatory diseases [3,4,5].

There are various diets in modern societies across ethnic populations including liquids and smoothies, convenient diets (canned food, high-school, and fast-food), Western diets (American, Mexican, Italian, and Mediterranean), and Eastern diets (Japanese, Chinese, and Korea) [2,25]. We validated essential nutrients with these domains of model diets [2,25], and healthy eating parameters with human diets across ethnic groups [3]. Our previous findings indicated that various modern diets might affect the accuracy in assessment of nutrients intakes, and heathy eating parameters [2,3,25]. Updated artificial intelligence (AI) machine-learning-based multivariate analytics, can be used to identify significant factors with improved accuracy [3,4,5]. The AI-based analytics with partition in iterations employs resampling with machine-learning operations for more accurate validations [5]. Thus, updated AI-based analytics with validation could be used to identify modifiable factors contributing to accurate prediction of healthy eating parameters for novel findings [2,25]. Therefore, the purpose of this study was to validate the predictors of HEI, GI, and GL across modern diets, in preparation for the precision-based e-health era.

## 2. Materials and Methods

### 2.1. Dietary Parameters and Indexes

We entered daily dietary data and assessed the nutrients of 131 diets in four domain groups (liquids, convenience, ethnic, and smoothie diets) consumed by diverse populations across modern social contexts [2,3,4,5,25]. Based on previous studies [2,3,25], validation with model diets in addition to diets taken by various ethnic groups, is needed for accurate estimate of nutrients. We grouped possible modern diets into (1) liquid diets; (2) convenient diets (canned food, high-school, and fast foods); (3) ethnic diets of Western (American, Mexican, Italian, and Mediterranean) and Eastern (Japanese, Chinese, and Korean), and (4) smoothies added to these diets [2,25]. All diets were processed using the Nutrition Data Systems for Research (NDSR) software [26,27,28]. Data entry and analyses were verified for accuracy by team members.

Healthy eating parameters were examined across various diets using HEI (HEI-2015) [29,30], GI [31], and GL [32]. HEI scored from 0 to 100 (0–50: poor, 51–80: moderate, >80: good [33]), which included food components of fruits, vegetables, grains, dairy, proteins, oils and nuts; with limiting saturated fats, sodium, and empty calories. Fruits (total and whole), vegetables (total and dark greens), and grains (total and whole) were each scored on a scale of 0–5. Dairy, proteins, oils and nuts, sodium, and saturated fats were scored on a scale of 0–10. Empty calories were scored on a scale of 0–20.

For diet quality, the amount of carbohydrates in the diets contributes to the glycemic Index (GI), calculated using the NDSR software based on daily diets. GI accounts carbohydrates in foods and the affected levels of blood sugar [3]. GI scored from 0 to 100, with ≤55 being good (with carbohydrates that were digested and metabolized slower for blood glucose and insulin); 56–69 being moderate; and ≥70 being poor [19,34,35].

GL was calculated using the NDSR software based on daily diets by multiplying GI by the amount of carbohydrates in grams (g) with servings of foods divided by 100. Daily GL could be divided by the number of meals needed to yield an acceptable GL per meal. A GL score of <10 was good, 11–19 being moderate, and ≥20 being poor [36]; thus, <20 being acceptable per meal. Therefore, we calculated daily GL values with 20 as a denominator to obtain the number of meals needed per day for each diet; and calculated normalized and standardized carbohydrates per meal by dividing the daily carbohydrates per diet with the median number of meals from all diets.

### 2.2. Data Analysis

Data analysis was performed using JMP^®^ Pro version 16.0.0 software ([37,38,39], SAS Institute, Cary City, NC, USA). The analytics and rationales were reported before [2,3,25] and the strengths are summarized in the following. For predictive analyses, JMP software presented logistic regression (LR) as a baseline default exploratory model to predict dependent variables in categorical values. Following LR, other AI machine-learning-based GR validation models (the Leave-One-Out model where least significant factors might be eliminated to avoid over-fitting, or Elastic Net models) might be chosen with validations for further confirmatory analysis. Conventional statistical procedures, including the baseline LR models, are restricted by the sample size in the datasets [5]. If the number of factor parameters to be estimated exceeds the degrees of freedom, based on the sample size, the conventional models could be highly unstable; whereas machine-learning based GR models minimizes the number of predictors in the model to avoid over-fitting. This machine-learning-based approach is superior to conventional statistics, including the LR models, that tend to yield an overfitted model [4,5]. The AI-based analytics employs partitions in iteration by resampling within the datasets with machine-learning operations [5]. By resampling, observed biases would be corrected by repeated analyses on random subsets of datasets [5]. We incorporated Elastic Net models for their capacity to operate complex datasets with multiple domains and many factor variables, balancing possible interactions from domain factors [37].

We utilized AI-based GR analytics to validate the predictions, with an 80/20 randomized split for training and validation sets for predictive modeling [38,39]; the final fittest model presented a lowest prediction error and minimized over-fitting [40]. For predictive modeling, we progressively examined significant factors per domains of HEI, caloric source, and various diets. With the Elastic Net validation models, we selected the fittest model based on precision criteria (Akaike Information Criterion with correction (AICc) the lower score, the fitter and better model; Misclassification Rate (MR): the lower the errors, the better; area under curve (AUC): the higher score, the more coverage and better) [41,42]. We further examined the graphical presentations on the prediction and interaction profilers to visualize potential interactions among the factors. To extend and continue a previous study [3], to identify the factors contributing to the health eating indices, we examined the prediction of GL in addition to HEI and GI. We further examined these indices with both measures of defined good quality scores (HEI > 80 and GI ≤ 55) and median scores.

## 3. Results

### 3.1. Healthy Eating Parameters

**Figure 1:**
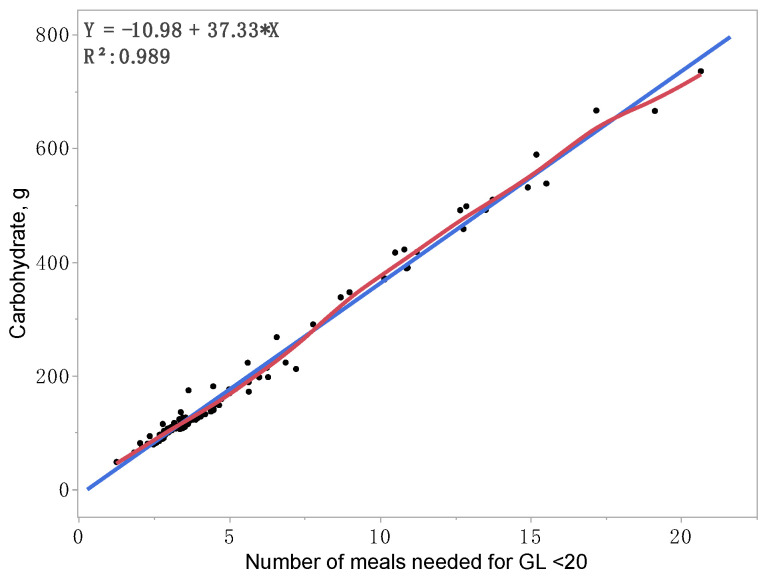
Prediction for carbohydrates per number of meals needed for glycemic load (GL) < 20. Blue line: linear fit, red line: curve fit.

The HEI, GI, and GL parameters are summarized for daily diets per four diet groups in Table 1 and all diets in Supplementary Table S1. Among the four diet groups, mean HEI scores ranked highest from 78.61 for smoothie diets, 65.28 for ethnic diets, 57.86 for convenient diets, to 48.78 for liquid diets (*p* < 0.0001, Table 1). All HEI parameters, including total fruits, whole fruits, vegetables, dark greens, total grains, whole grains, dairy, proteins, oils and nuts, saturated fats, sodium, and empty calories, were significantly different among the four diet groups (*p* < 0.0001). None of the liquid and convenient diets presented a good HEI score of >80, whereas 1.4% (1/71) of the ethnic diets and 50% (11/22) of the smoothie diets illustrated a good HEI score. Mean GI scores ranked highest from 59.86 for convenient diets, 58.88 for ethnic diets, 56.38 for liquid diets, to 54.52 for smoothie diets (*p* < 0.0001). For a good GI score of ≤55, 3.33% (1/30) of the convenient diets, 12.9% (9/71) of the ethnic diets, 25% (2/8) of the liquid diets, and 63.6% (14/22) of the smoothie diets met the criteria. The mean daily GL (GI x carbohydrates/100) ranked highest from 240.8 for smoothie diets, 89.20 for convenient diets, 74.30 for liquid diets, to 68.69 for ethnic diets (*p* < 0.0001). To reach a good GL of <20 per meal, the daily number of meals with smoothie diets would need to be an average of 12.04 times, 4.46 for convenient diets, 3.71 for liquid diets, and 3.43 for ethnic diets (*p* < 0.0001, median = 3.59 meals daily). Contributing to GL, mean carbohydrates ranked highest from 442.2 g for smoothie diets, 148.7 g for convenient diets, 135.6 g for liquid diets, to 117.4 g for ethnic diets (*p* < 0.0001). Standardized mean carbohydrates (divided by the median number of meals 3.59 to reach a good GL of <20 per meal) ranked highest from 36.83 g for smoothies, 35.73 g for liquids, 34.23 g for ethnic, and 33.50 g for convenient diets (*p* = 0.0003, median = 33.95 g). To further validate the prediction for a good GL of <20 per meal, a regression coefficient of 37.33 g carbohydrates per meal was derived across all diets (Figure 1). Additional details for all diets per groups of diets on HEI, GI, GL, number of meals to reach acceptable GL, carbohydrates, and standardized carbohydrates/meals (divided by the median number of meals 3.59 to reach a good GL of <20 per meal) are listed in Supplementary Table S1.

### 3.2. Predictive Modeling for Healthy Eating Parameters

In testing predictive modeling of HEI, GI, and GL, we progressively included related factors per domains of HEI, caloric sources, and various diets. Final predictive models of HEL, GI, and GL were determined based on the fittest models with the lowest AICc, least misclassification, and highest AUC for coverage (Table S2: progression for HEI, Table S3: GI, Table S4: GL, Table S5: carbohydrates, and Table S6: standardized carbohydrates per median number of meals needed for GL < 20). For HEI (>80 as a good score), three factors of HEI, including whole fruits, whole grains, and empty calories, were most significant for the fittest model (*p* < 0.0001; AICc: 9.78, misclassification: 0; Table 2: baseline LR model on the left panel and GR validation model on the right panel; AUC: 1.00: Figure S1). The inclusion of factors from the other two domains (caloric and diets) did not yield a better or fitter model on HEI (>80) prediction (Table S2). Additionally, prediction on HEI median score (≥64.4; AICc: 29.87, misclassification: 0.2, AUC: 0.94) did not reach a better model, while total fruits, dark greens, and canned foods were significant factors (Table S2).

For predictive modeling of GI, significant predictors for GI (≤55 as good quality score) included total fruits, carbohydrates, and Mexican diets, which presented one factor from each of the HEI, caloric, and diet domains (Table 3). Additionally, significant predictors for GI median score (≤59) included total fruits from the HEI domain and two diets of Mexican and Chinese (Table 3, Table S3). Thus, both final models of GI presented very similar AICc; for precise fitness; compared to GI median score (≤59), GI (≤55) had slightly (0.03) lower and better AICc (33.63 vs. 33.66) but higher misclassification (0.23 versus 0.13), and lower AUC (0.84 versus 0.89, Figure S2).

For predictive modeling of GL (≤71.8 median score), the unique significant predictor for the fittest model was carbohydrates (*p* < 0.0001; Table 4, Table S4, Figure S3). As carbohydrates was the common predictor for GI and GL, we further examined the factors from other domains that contributed to the source of carbohydrates (Table S5) and standardized carbohydrates per median number of meals across all diets (Table S6).

To reach an acceptable GL < 20 per meal, we calculated and derived standardized carbohydrates (≤33.95 g) per median number of meals across all diets (3.59 meals needed daily). Significant predictors included three diets: canned food, Mexican, and smoothie diets (Table 5, *p* < 0.01). Additionally, total fruits and whole grains from the HEI domain and Mexican diets could be considered in looking for the sources of carbohydrates (Table 5, *p* < 0.01; Table S6, Figure S4). Model including two factors from the HEI domain (total fruits, whole grains) with Mexican diets presented better AUC:(0.88 versus 0.8125); however, slightly higher AICc (36.33 versus 35.09) and higher misclassification rate (0.3 versus 0.2).

## 4. Discussion

We presented a novel study, using AI machine-learning-based analytics with added validation criteria for enhanced accuracy, to illustrate the predictors of healthy eating parameters measured by HEI, GI, and GL by including various modern diets. Consistent with a previous study [3], this study confirmed predictors of HEI 80 included whole fruits and whole grains [3], with empty calories as an additional predictor. Predictors of GI included total fruits, carbohydrates, and Mexican diets. The predictor of GL included carbohydrates. Predictors to reach an acceptable GL < 20 with carbohydrates per meal included three diet types (canned food, Mexican, and smoothies), considering total fruits and whole grains in the diets being the source of carbohydrates. To summarize, the common predictors included total fruits for HEI and GI; carbohydrates for GI and GL; and Mexican diets for GI and carbohydrates per meal to reach an acceptable GL < 20.

For additional novel findings as strengths of this study, we demonstrated GI, GL, and related carbohydrates per day and per meal. To reach a good GL of <20 per meal, the daily number of meals ranged between 3.43 for ethnic diets and 12.04 for smoothie diets (median = 3.59). By accounting for the number of meals (median = 3.59) to reach a good GL of <20 per meal, standardized mean carbohydrates ranked between 33.5 g for convenient diets to 36.83 g for smoothies (median = 33.95), with a regression coefficient of 37.33 g (Figure 1). However, there are limitations to the accuracy of GI and GL due to factors such as food ripeness, processing, nutrient interactions, and cooking method [43]. Current nutritional assessment tools including diaries do not contain adjustments based on food ripeness, processing, nutrient interactions, and cooking method, except adjustment on fat content using the Food Frequency Questionnaire that we have integrated in this study [25]. Including modern diets as a factor (convenient and ethnic diets) in this study was an attempt to consider different cooking methods. These limitations may have implications for our findings. Further studies are needed to better understand the impact of these factors on the accuracy of GI, GL, and carbohydrates related factors.

Based on the benefits of low GI diets, professional organizations, including ADA, medical, and health professionals, currently suggest limiting the amount of carbohydrates per meal to 45–60 g [19,20,21,22,23,24]. However, scientific data is lacking to solidify the amount of carbohydrates needed to reach for acceptable GL of <20 per meal. As a beginning effort, we calculated the amount of carbohydrates to reach an acceptable GL < 20 as 33.95 g per median number of meals (3.59 meals daily) across all diets. The source of carbohydrates in the HEI domain included total fruits, vegetables, whole grains, and empty calories in relation to GI, GL, and GL < 20.

Adding smoothies into the diet can supplement essential nutrients; however, carbohydrates could be increased in the diet to increase GL. To reach an acceptable GL < 20 per meal, the number of meals per day would need to be increased. Other diets that presented a higher median number of meals (median average: 3.59 meals across all diets) included high-school (5.75), fast-food (4.48), Korean (4.30), Chinese (3.93), and liquids (3.71). Additionally, Mexican diets was the common predictor for GI and carbohydrates per meal to reach acceptable GL < 20. Specifically, the number of meals might need to be increased daily for diets that contain a higher amount of carbohydrates to reach an acceptable GL < 20 per meal. Professional organizations recommended counting carbohydrates (45–60 g per meal) to manage GL by monitoring carbohydrate sources [19,20,21]. To reach an acceptable GL < 20 per meal, we calculated the associated median amount of carbohydrates per meal as 33.95 g and a regression coefficient as 37.33 (Figure 1), which are lower than the 45 g recommended for people with diabetes [19,20,21,44,45]. Further studies are needed to account for the source and the amount of carbohydrates per meal to manage GL for disease prevention.

In summary, assessing healthy eating parameters with HEI, GI, and GL can be helpful in the e-health era to assist various populations by mindfully consuming adequate nutrients for healthy living. Following this research effort, further studies are needed to include various modern diets to validate these findings further [46]. Balanced diets, including all elements of the HEI domain, are necessary to supply essential nutrients [47,48], and counting carbohydrates and sources of carbohydrates might also be critical to control GL and reduce disease risks [49]. For personalized nutrition in the precision-based e-health era, healthy eating habits with counting sources and amounts of nutrients are essential to improve health outcomes for various populations [4].

## Figures and Tables

**Table 1:** Healthy eating parameters per four groups of diets per daily meals (*N* = 131).

Parameters, Units (Units, Max Score)	M ± SD M ± SD	Liquid N = 8	Convenient N = 30	Ethnic N = 71	Smoothie N = 22	*p* (*F*)
Total Fruits, cup	Intake	2.27 ± 1.70	0.39 ± 0.40	0.86 ± 0.94	5.88 ± 1.60	<0.0001
(>0.8 cup, 5)	Score	5 ± 0	2.09 ± 1.53	3.13 ± 1.29	5 ± 0	^S > L E C; L > E C^
Whole Fruits, cup	Intake	1.49 ± 2.17	0.11± 0.12	0.69 ± 0.89	5.53 ± 1.52	<0.0001
(>0.4 cup, 5)	Score	2.50 ± 2.67	1.33 ± 1.49	3.87 ± 1.03	5 ± 0	^S > L E C; L > C^
Vegetables, cup	Intake	1.71 ± 3.17	0.89 ± 0.50	1.07 ± 0.80	7.40 ± 2.09	<0.0001
(>1.1 cup, 5)	Score	1.25 ± 2.31	3.47 ± 1.10	4.00 ± 1.02	5 ± 0	^S > L E C^
Dark greens, cup	Intake	0.86 ±1.62	0.12 ± 0.29	0.41 ± 0.44	3.73 ± 1.01	<0.0001
(>0.4 cup, 5)	Score	1.25 ± 2.31	1.04 ± 1.18	3.69 ± 1.17	5 ± 0	^S > L E C; L > C^
Total Grains, oz	Intake	0.00 ± 0.00	2.28 ± 1.46	2.65 ± 1.72	4.27 ± 2.79	<0.0001
(>3 oz., 5)	Score	0.00 ± 0.00	3.24 ± 1.43	3.86 ± 1.00	4.33 ± 1.20	^S > E C L; E C > L^
Whole Grains, oz	Intake	0.00 ± 0.00	0.27 ± 0.16	0.51 ± 0.46	0.92 ± 0.61	<0.0001
(>1.5 oz., 5)	Score	0.00 ± 0.00	0.88 ± 0.52	1.58 ± 1.02	2.80 ± 1.61	^S > E C L; E > C L^
Dairy, cup	Intake	0.88 ± 0.40	0.60 ± 0.19	0.52 ± 0.21	0.82 ± 0.71	0.0014
(>1.3 cup, 10)	Score	6.72 ± 3.08	4.61 ± 1.48	4.02 ± 1.60	5.29 ± 3.50	^L S > E^
Proteins, oz	Intake	0.00 ± 0.00	4.77 ± 2.95	3.87 ± 1.45	9.15 ± 6.16	<0.0001
(>2.5 oz., 10)	Score	0.00 ± 0.00	9.36 ± 1.07	9.16 ± 1.46	9.49 ± 1.47	^S > C E L; C E > L^
Oils and Nuts, g	Intake	0.06 ± 0.08	12.77 ± 10.25	4.47 ± 3.12	21.80 ± 28.97	<0.0001
(>12 g, 10)	Score	0.05 ± 0.07	7.35 ± 3.81	3.51 ± 2.24	7.33 ± 3.44	^S > E L; C > E^
Saturated Fats, % calorie	Intake	1.57± 1.78	17.12 ± 6.13	10.27 ± 5.79	23.07 ± 17.37	<0.0001
(<8% calorie, 10)	Score	8.91 ± 2.08	5.42 ± 2.10	6.63 ± 2.67	9.12 ± 1.81	^S > E L; C > E L; E > L^
Sodium, g	Intake	2.45 ± 1.82	2.25 ± 0.84	2.62 ± 1.80	5.81 ± 2.45	<0.0001
(<1.1 g, 10)	Score	3.75 ± 5.18	1.39 ± 1.67	1.31 ± 2.13	0 ± 0	^S > E L^
Empty Calories, calorie	Intake	203.50 ± 0.00	283.6 ± 169.5	61.89 ± 39.19	171.50 ± 179.2	<0.0001
(<19% calorie, 20)	Score	19.34 ± 1.86	17.14 ± 4.08	20.00 ± 0.00	20 ± 0	^C > S E; L S > E^
Healthy Eating Index Score		48.78 ± 6.37	57.86 ± 4.90	65.28 ± 5.17	78.61 ± 7.43	<0.0001
>80 (good)		0 (0%)	0 (0%)	1 (1.4%)	11 (50%)	^S > E C L; E > C L; C > L^
≥64.4 (median distribution)		0 (0%)	3 (10%)	38 (53.5%)	21 (95.5%)	
Glycemic Index Score		56.38 ± 4.96	59.86 ± 3.06	58.88 ± 4.82	54.52 ± 3.72	<0.0001
≤55 (low and good)		2 (25.0%)	1 (3.33%)	9 (12.9%)	14 (63.6%)	^C E > S^
≤59 (median distribution)		3 (27.3%)	12 (40%)	20 (28.2%)	21 (95.5%)	
Glycemic Load (GI x Carbohydrate/100)		74.30 ± 39.47	89.20 ± 24.87	68.69 ± 31.71	240.8 ± 75.77	<0.0001
≤71.8 (median distribution)		4 (50%)	9 (30%)	53 (74.6%)	0 (0%)	^S > E L C^
≥20 (high)		8 (100%)	30 (100%)	71 (100%)	22 (100%)	
Number of Meals Needed for GL < 20		3.71 ± 1.97	4.46 ± 1.24	3.43 ± 1.59	12.04 ± 3.79	<0.0001
≤3.59 (median distribution)		4 (50%)	9 (30%)	53 (74.6%)	0 (0%)	^S > E L C^
Carbohydrates, g		135.6 ± 81.01	148.7 ± 40.40	117.4 ± 55.73	444.2 ± 140.3	<0.0001
≤123.4 g (median distribution)		4 (50%)	10 (33.3%)	52 (73.2%)	0 (0%)	^S > E L C^
Carbohydrates/Meals (GL < 20), g		35.73 ± 3.30	33.50 ± 1.71	34.23 ± 3.31	36.83 ± 2.23	0.0003
≤33.95 g (median distribution)		5 (62.5%)	17 (56.7%)	43 (60.6%)	1 (4.5%)	^S > C E^

*Note*. M: mean, SD: standard deviation, oz.: ounce, g: gram; L: Liquid, C: Convenient, E: Ethnic, S: Smoothie, *p* < 0.05 noted for significant post-hoc tests.

**Table 2:** Predictors of Healthy Eating Index (>80).

Parameters, Median Units	Logistic Regression Original Model		Generalized Regression Elastic Net Validation	
	Estimate (95% CI)	*p* (*χ*^2^)	Estimate (95% CI)	*p* (*χ*^2^)
(Intercept)	−12.46 (−289.1–264.2)	0.9296	−9.35 (−9.90–−8.81)	<0.0001
Whole Fruits, ≥0.33 cup	−14.56 (−301.0–271.9)	0.9206	−11.40 (−13.54–−9.26)	<0.0001
Whole Grains, ≥0.41 oz	14.10 (−292.0–263.7)	0.9208	−11.02 (−13.21–−8.83)	<0.0001
Empty Calories, ≤88.87 calorie	−14.86 (−261.8–291.5)	0.9161	11.75 (9.64–13.86)	<0.0001
MR	0.00		0.00	
AICc	9.77		9.78	
AUC	1.00		1.00	

*Note*. MR: misclassification rate; AICc: Akaike’s information criterion with corrections; AUC: area under the curve; CI: confidence interval.

**Table 3:** Predictors of Glycemic Index.

Parameters, Median Units	Logistic Regression Original Model		Generalized Regression Elastic Net Validation	
	Estimate (95% CI)	*p* (*χ*^2^)	Estimate (95% CI)	*p* (*χ*^2^)
**GI** **≤ 55**				
(Intercept)	11.89 (−131.97–155.74)	0.8713	9.68 (7.30–12.05)	<0.0001
Total Fruits, ≥0.43 cup	−11.74 (−155.58–−132.11)	0.8729	−9.53 (−10.27–−8.78)	<0.0001
Carbohydrates, ≥123.4 g	−2.06 (−3.44–−0.67)	0.0037	−2.06 (−3.42–−0.69)	0.0032
Mexican Diets	−11.83 (−155.69–132.02)	0.8719	−9.63 (−11.91–−7.35)	<0.0001
MR	0.23		0.23	
AICc	33.63		33.63	
AUC	0.84		0.84	
**GI ≤ 59 (median)**				
(Intercept)	1.97 (−155.9–159.8)	0.9805	1.84 (0.14–3.54)	0.0336
Total Fruits, ≥0.43 cup	−1.47 (−2.38–−0.56)	0.0016	−1.47 (−2.38–−0.56)	0.0016
Mexican Diets	−10.83 (−127.6–106.0)	0.8558	−9.92 (−10.99–−8.86)	<0.0001
Chinese Diets	9.46 (−96.73–115.7)	0.8614	8.69 (7.63–9.74)	<0.0001
MR	0.13		0.13	
AICc	33.66		33.66	
AUC	0.89		0.89	

*Note*. MR: misclassification rate; AICc: Akaike’s information criterion with corrections; AUC: Area under the curve; CI: confidence interval.

**Table 4:** Predictors of Glycemic Load (≤71.8).

Parameters, Median Units	Logistic Regression with Validation		Generalized Regression Elastic Net Validation	
	Estimate (95% CI)	*p* (*χ*^2^)	Estimate (95% CI)	*p* (*χ*^2^)
(Intercept)	−2.48 (−3.50–−1.46)	<0.0001	−1.96 (−2.63–−1.28)	<0.0001
Carbohydrates, ≥123.4 g	4.66 (3.28–6.04)	<0.0001	3.67 (2.72–4.62)	<0.0001
MR	0.13		0.13	
AICc	29.33		28.53	
AUC	0.8733		0.8733	

*Note*. MR: misclassification rate; AICc: Akaike’s information criterion with corrections; AUC: area under the curve; CI: confidence interval.

**Table 5:** Predictors of standardized carbohydrates per median number of meals (≤33.95 g) needed for glycemic load (GL) < 20.

Parameters, Median Units	Logistic Regression with Validation		Generalized Regression Elastic Net Validation	
	Estimate (95% CI)	*p* (*χ*^2^)	Estimate (95% CI)	*p* (*χ*^2^)
**3 Diet Factors (Final Model)**				
(Intercept)	−17.38 (−183.4–148.6)	0.8374	−15.81 (−19.00–−12.61)	<0.0001
Canned Food Diets	1.41 (0.45–2.36)	0.0082	2.91 (0.75–5.07)	0.0082
Mexican Diets	2.91 (0.75–5.07)	0.8907	10.06 (9.10–11.02)	<0.0001
Smoothie Diets	3.80 (1.72–5.89)	0.0004	3.80 (1.72–5.88)	0.0004
MR	0.20		0.20	
AICc	35.09		35.09	
AUC	0.8125		0.8125	
**2 HEI and 1 Diet Factors**				
(Intercept)	−12.26 (−178.2–153.8)	0.8849	−10.85 (−12.09–−9.61)	<0.0001
Total Fruits, ≥0.43 cup	1.59 (0.65–2.53)	0.0009	1.59 (0.65–2.54)	0.0009
Whole Grains, ≥0.41 oz	1.41 (0.45–2.36)	0.0039	1.41 (0.46–2.36)	0.0037
Mexican Diets	10.89 (−115.1–176.9)	0.8977	9.48 (8.29–10.66)	<0.0001
MR	0.30		0.30	
AICc	36.33		36.33	
AUC	0.88		0.88	

*Note*. MR: misclassification rate; AICc: Akaike’s information criterion with corrections; AUC: area under the curve; CI: confidence interval.

## Supplementary Materials

The following are available online at https://www.mdpi.com/article/10.3390/nu15051263/s1, Supplementary Table S1. Healthy eating parameters for all diets per day (N = 131), Supplementary Table S2. Progression on selecting significant factors contributing to Health Eating Index (HEI), Supplementary Table S3. Progression on selecting significant factors contributing to glycemic index (GI), Supplementary Table S4. Progression on selecting significant factors contributing to glycemic load (GL), Supplementary Table S5. Progression on selecting significant factors contributing to carbohydrates, Supplementary Table S6. Progression on selecting significant factors contributing to standardized carbohydrates per median number of meals (≤33.95 g) needed for glycemic load (GL) < 20; Supplementary Figure S1. Predictors of Healthy Eating Index (80), including 3 HEI factors (whole fruit, whole grains, and empty calories): Area under the receiver operating characteristic curve (AUC) for baseline (a) logistic regression model; (b) Elastic Net with Akaike’s information criteria with correction (AICc) validation model, Supplementary Figure S2. Predictors of Glycemic Index (55), including 1 HEI (total fruit), 1 Caloric (carbohydrate), and 1 Diet Factors (Mexican diet): Area under the receiver operating characteristic curve (AUC) for baseline (a) logistic regression model; (b) Elastic Net with Akaike’s information criteria with correction (AICc) validation model, Supplementary Figure S3. Predictors of Glycemic Load (71.8), Carbohydrate: Area under the receiver operating characteristic curve (AUC) for baseline (a) logistic regression model; (b) Elastic Net with Akaike’s information criteria with correction (AICc) validation model, Supplementary Figure S4. Predictors of standardized carbohydrates per number of meals needed for GL < 20, (≤33.95 g), including 3 Diet Factors (canned food, Mexican, and smoothie diets): Area under the receiver operating characteristic curve (AUC) for baseline (a) logistic regression model; (b) Elastic Net with Akaike’s information criteria with correction (AICc) validation model.
