# Supplementary material for: Validating Healthy Eating Index, Glycemic Index, and Glycemic Load with Modern Diets for E-Health Era
Source: Nutrients. 2023 Mar 3;15(5):1263. doi: 10.3390/nu15051263 (PMC10005628; doi:10.3390/nu15051263)
Supplement: Supplementary file 1 [file nutrients-15-01263-s001.zip › nutrients-2195962-supplementary.pdf]

Supplementary Table S1. Healthy eating parameters for all diets per day (N=131).

Diets (n) M ± SD	Healthy Eating Index	Glycemic Index	Glycemic Load	n meals (GL <20)	Carbohydrates g	Carbohydrates / meals (GL <20), g	Calories kcal	Protein g	Total Fat g
Liquid (8)	48.78 ± 6.37	56.38 ± 4.96	74.30 ± 39.47	3.71 ± 1.97	135.6 ± 81.01	35.73 ± 3.30	697.3 ± 324.3	24.19 ± 9.89	10.61 ± 5.15
Convenient (30)	57.86 ± 4.90	59.86 ± 3.06	89.20 ± 24.87	4.46 ± 1.24	148.7 ± 40.40	33.50 ± 1.71	1,319 ± 375.1	40.77 ± 17.67	64.57 ± 25.15
Canned Food (10)	54.60 ± 1.99	56.98 ± 1.82	63.06 ± 5.78	3.15 ± 0.29	110.6 ± 9.16	35.13 ± 1.09	891.9 ± 52.95	29.00 ± 4.11	38.21 ± 8.34
High-school (10)	59.02 ± 4.56	60.32 ± 2.14	115.0 ± 17.50	5.75 ± 0.88	191.2 ± 31.89	33.20 ± 1.18	1,644 ± 299.1	61.88 ± 15.03	72.86 ± 15.03
Fast-food (10)	59.98 ± 5.86	62.27 ± 2.53	89.53 ± 12.42	4.48 ± 0.62	144.2 ± 22.89	32.17 ± 1.36	1,421 ± 172.2	31.42 ± 4.12	82.63 ± 23.09
Ethnic (71)	65.28 ± 5.17	58.88 ± 4.82	68.69 ± 31.71	3.43 ± 1.59	117.4 ± 55.73	34.23 ± 3.31	955.2 ± 388.1	44.17 ± 16.51	34.90 ± 14.74
Western (38)	64.82 ± 3.22	57.29 ± 4.15	63.47 ± 11.28	3.17 ± 0.56	111.7 ± 23.77	35.13 ± 3.22	950.4 ± 225.1	43.14 ± 8.07	37.13 ± 12.71
American (9)	67.24 ± 2.94	59.12 ± 1.88	67.71 ± 11.82	3.39 ± 0.59	114.4 ± 18.72	33.86 ± 1.10	873.6 ± 92.82	38.33 ± 5.01	29.65 ± 1.95
Mexican (10)	61.75 ± 2.89	52.92 ± 5.90	66.64 ± 6.04	3.33 ± 0.30	128.3 ± 25.53	38.30 ± 5.02	1,161 ± 267.9	48.80 ± 4.66	50.65 ± 16.59
Italian (10)	64.54 ± 0.87	58.79 ± 0.90	68.44 ± 5.91	3.42 ± 0.30	116.4 ± 10.07	34.03 ± 0.53	1,030 ± 49.80	50.01 ± 2.12	39.73 ± 1.73
Mediterranean (9)	66.11 ± 2.94	58.66 ± 1.86	50.18 ± 10.31	2.51 ± 0.52	85.29 ± 15.87	34.12 ± 1.05	704.2 ± 62.07	34.05 ± 5.35	26.70 ± 2.79
Eastern (33)	65.80 ± 6.78	60.70 ± 4.95	74.71 ± 44.53	3.74 ± 2.23	124.0 ± 77.82	33.20 ± 3.14	960.8 ± 520.4	45.36 ± 22.76	32.34 ± 16.60
Japanese (10)	58.97 ± 0.57	62.46 ± 0.42	56.14 ± 7.29	2.81 ± 0.36	89.92 ± 12.00	32.02 ± 0.22	769.4 ± 45.07	40.54 ± 7.28	27.57 ± 1.61
Chinese (10)	65.75 ± 2.66	63.25 ± 1.56	78.58 ± 9.73	3.93 ± 0.49	124.1 ± 14.00	31.64 ± 0.81	1,048 ± 48.15	50.10 ± 3.39	40.58 ± 2.32
Korean (13)	71.11 ± 6.89	57.38 ± 6.59	86.02 ± 68.83	4.30 ± 3.44	150.1 ± 119.1	35.30 ± 4.22	1,041 ± 821.7	45.42 ± 35.99	29.67 ± 25.41
Smoothie (22)	78.61 ± 7.43	54.52 ± 3.72	240.8 ± 75.77	12.04 ± 3.79	444.2 ± 140.3	36.83 ± 2.23	2,802 ± 1,137	103.9 ± 44.38	80.59 ± 52.41

Note. M: mean; SD: standard deviation; g: gram; kcal: calories.

Supplementary Table S2. Progression on selecting significant factors contributing to Health Eating Index (HEI).

Parameters, median units	Logistic Regression with Validation				Generalized Regression Elastic Net Validation			
	p (χ^2)	MR	AICc	AUC	p (χ^2)	MR	AICc	AUC
**HEI ≥80**								
**12 HEI Factors**								
(Intercept)	0.9859	0.00	48.75	1.00	0.3137	0.00	42.36	1.00
Whole Fruits, ≥0.33 cup	0.9515				<0.0001			
Dark Greens, ≥0.27 cup	0.9518				<0.0001			
Total Grains, ≥2.23 oz	0.9993				0.0352			
Whole Grains, ≥0.41 oz	0.9828				<0.0001			
Dairy ≥0.56, cup	0.8814				<0.0001			
Proteins, ≥3.60 oz	0.9793				0.0024			
Empty Calories, ≤88.87 calorie	0.9337				<0.0001			
Total Fruits, ≥0.43 cup	0.9935				0.2875			
Vegetables, ≥1 cup	0.9890				1.0000			
Oils and nuts, ≥3.51 g	0.9885				0.2976			
Sodium, ≤2.17 g	0.9666				0.6188			
Saturated Fats, ≤10.32 % calorie	0.9962				0.8361			
**7 HEI Factors**								
(Intercept)	0.9922	0.00	22.88	1.00	<0.0001	0.00	22.87	1.00
Whole Fruits, ≥0.33 cup	0.9598				<0.0001			
Dark Greens, ≥0.27 cup	0.9361				<0.0001			
Total Grains, ≥2.23 oz	0.9875				<0.0001			
Whole Grains, ≥0.41 oz	0.9112				<0.0001			
Dairy, ≥0.56 cup	0.8898				<0.0001			
Proteins, ≥3.60 oz	0.9951				0.0026			
Empty Calories, ≤88.87 calorie	0.9679				<0.0001			
**3 HEI Factors (Final model, Table 3)**								
(Intercept)	0.9296	0.00	9.77	1.00	<0.0001	0.00	9.78	1.00
Whole Fruits, ≥0.33 cup	0.9206				<0.0001			
Whole Grains, ≥0.41 oz	0.9208				<0.0001			
Empty Calories, ≤88.87 calorie	0.9161				<0.0001			
**3 Caloric Factors**								
(Intercept)	0.1491	0.03	18.00	0.88	0.1489	0.03	18.00	0.88
Carbohydrates, ≥123.4 g	0.9314				<0.0001			
Protein, ≥43.8 g	0.9272				<0.0001			
Fat, ≥39 g	0.9275				<0.0001			
**2 Diet Factors**								
(Intercept)	0.8852	0.10	13.60	0.95	<0.0001	0.10	13.60	0.95
Korean Diets	0.8875				<0.0001			
Smoothie Diets	0.8624				<0.0001			
**3 HEI, 1 Caloric, and 1 Diet Factors**								
(Intercept)	0.9797	0.00	15.84	1.00	0.0011	0.00	15.85	1.00
Whole Fruits, ≥0.33 cup	0.9237				<0.0001			
Whole Grains, ≥0.41 oz	0.9304				<0.0001			
Empty Calories, ≤88.87 calorie	0.9564				<0.0001			
Fat, ≥39 g	0.9930				0.0056			
Korean Diets	0.9147				0.0012			

Continue Table S2

Parameters, median units	Logistic Regression with Validation				Generalized Regression Elastic Net Validation			
	p (χ^2)	MR	AICc	AUC	p (χ^2)	MR	AICc	AUC
**HEI ≥64.4 (median)**								
**12 HEI Factors**								
(Intercept)	0.0177	0.17	94.02	0.91	<0.0001	0.23	67.81	0.88
Total Fruits, ≥0.43 cup	0.0485				0.0002			
Dark Greens, ≥0.27 cup	0.0221				0.0001			
Total Grains, ≥2.23 oz	0.0194				<0.0001			
Whole Grains, ≥0.41 oz	0.0579				0.0488			
Dairy, ≥0.56 cup	0.0684				0.0221			
Saturated Fats, ≤10.32 % calorie	0.1116				0.0298			
Whole Fruits, ≥0.33 cup	0.0485				0.1329			
Vegetables, ≥1 cup	0.1098				0.7210			
Proteins, ≥3.60 oz	0.0677				0.1871			
Oils and nuts, ≥3.51 g	0.9902				1.0000			
Sodium, ≤2.17 g	0.9076				0.8301			
Empty Calories, ≤88.87 calorie	0.9403				0.7843			
**3 HEI Factors**								
(Intercept)	<0.0001	0.13	34.16	0.92	<0.0001	0.13	34.16	0.92
Total Fruits, ≥0.43 cup	0.0361				0.0243			
Dark Greens, ≥0.27 cup	<0.0001				<0.0001			
Whole Grains, ≥0.41 oz	0.0031				0.0018			
**3 Diet Factors**								
(Intercept)	0.8964	0.40	40.62	0.73	<0.0001	0.40	40.62	0.73
Canned Food Diets	0.9163				<0.0001			
Japanese Diets	0.9163				<0.0001			
Smoothie Diets	0.0068				0.0068			
**2 HEI and 1 Diet Factors (Final model)**								
(Intercept)	0.9094	0.20	29.86	0.94	<0.0001	0.20	29.87	0.94
Total Fruits, ≥0.43 cup	0.0006				0.0008			
Dark Greens, ≥0.27 cup	<0.0001				<0.0001			
Canned Food Diets	0.8845				<0.0001			

Note. MR: Misclassification rate; AICc: Akaike's information criterion with corrections; AUC: Area under the curve; HEI: Healthy Eating Index; Protein: oz from HEI, g from caloric factors. (none of caloric factors were significant for HEI median ≥64.4 prediction)

Supplementary Table S3. Progression on selecting significant factors contributing to glycemic index (GI).

Parameters, median units	Logistic Regression with Validation				Generalized Regression Elastic Net Validation			
	p (χ^2)	MR	AICc	AUC	p (χ^2)	MR	AICc	AUC
**GI ≤55**								
**2 HEI Factors**								
(Intercept)	0.8377	0.27	37.74	0.73	0.2629	0.23	35.50	0.73
Total Fruits, ≥0.43 cup	0.0012				0.0024			
Vegetables, ≥1 cup	0.0044				0.0013			
**1 Caloric Factor**								
(Intercept)	0.0146	0.23	36.36	0.64	0.0037	0.23	35.60	0.64
Carbohydrates, ≥123.4 g	0.0025				0.0171			
**5 Diet Factors**								
(Intercept)	0.8615	0.23	36.25	0.86	<0.0001	0.23	36.26	0.86
Liquids Diets	0.8614				<0.0001			
Canned Food Diets	0.8738				<0.0001			
Mexican Diets	0.8587				<0.0001			
Smoothie Diets	0.8420				<0.0001			
Korean Diets	0.8605				<0.0001			
**1 HEI, 1 Caloric, and 1 Diet Factors (<u>Final Model)</u>**								
(Intercept)	0.8713	0.23	33.63	0.84	<0.0001	0.23	33.63	0.84
Total Fruits, ≥0.43 cup	0.8729				<0.0001			
Carbohydrates, ≥123.4 g	0.0037				0.0032			
Mexican Diets	0.8719				<0.0001			
**GI ≤59 (median)**								
**2 HEI Factors)**								
(Intercept)	0.0003	0.37	39.72	0.77	0.0006	0.37	39.72	0.77
Total Fruits, ≥0.43 cup	0.0026				0.0026			
Whole Grains, ≥0.41 oz	0.0004				0.0005			
**5 Diet Factors**								
(Intercept)	0.9780	0.23	37.95	0.87	0.2130	0.23	37.95	0.87
Canned Food Diets	0.0251				0.0251			
Mexican Diets	0.8947				<0.0001			
Chinese Diets	0.8971				<0.0001			
Japanese Diets	0.8900				<0.0001			
Smoothie Diets	0.0016				0.0016			
**1 HEI and 2 Diet Factors (Final Model)**								
(Intercept)	0.9805	0.13	33.66	0.89	0.0336	0.13	33.66	0.89
Total Fruits, ≥0.43 cup	0.0016				0.0016			
Mexican Diets	0.8558				<0.0001			
Chinese Diets	0.8614				<0.0001			

Note. MR: Misclassification rate; AICc: Akaike's information criterion with corrections; AUC: Area under the curve; GI: Glycemic Index; HEI: Healthy Eating Index.

Supplementary Table S4. Progression on selecting significant factors contributing to glycemic load (GL).

Parameters, median units	Logistic Regression with Validation				Generalized Regression Elastic Net Validation			
	p **(χ^2)**	**MR**	**AICc**	**AUC**	***p*** **(χ^2)**	**MR**	**AICc**	**AUC**
**GL ≤71.8 (median)**								
**3 HEI Factors**								
(Intercept)	0.0918	0.33	43.57	0.78	0.1463	0.33	42.93	0.78
Vegetables, ≥1 cup	0.0064				0.0169			
Sodium, ≤2.17 g	0.0197				0.0324			
Empty Calories, ≤88.87 calorie	0.0003				0.0007			
**1 Caloric Factor (Final Model)**								
(Intercept)	<0.0001	0.13	29.33	0.87	<0.0001	0.13	28.53	0.87
Carbohydrates, ≥123.4 g	<0.0001				<0.0001			
**3 Diet Factors**								
(Intercept)	0.8743	0.13	31.23	0.85	<0.0001	0.13	31.24	0.85
High school Diets	0.9353				<0.0001			
Fast food Diets	0.9309				<0.0001			
Smoothie Diets	0.8965				<0.0001			
**1 Caloric and 3 Diet Factors**								
(Intercept)	0.8840	0.13	30.85	0.93	<0.0001	0.13	30.85	0.93
Carbohydrates, ≥123.4 g	<0.0001				<0.0001			
High School Diets	0.9440				<0.0001			
Fast food Diets	0.9401				<0.0001			
Smoothie Diets	0.9103				<0.0001			

Note. MR: Misclassification rate; AICc: Akaike's information criterion with corrections; AUC: Area under the curve; GL: Glycemic Load; HEI: Healthy Eating Index.

Supplementary Table S5. Progression on selecting significant factors contributing to carbohydrates.

Parameters, median units	Logistic Regression with Validation				Generalized Regression Elastic Net Validation			
	p (χ^2)	MR	AICc	AUC	p (χ^2)	MR	AICc	AUC
**Carbohydrate ≤123.4 g (median)**								
**4 HEI Factors**								
(Intercept)	0.6177	0.20	36.87	0.87	0.6416	0.20	36.87	0.87
Vegetables, ≥1 cup	0.0028				0.0004			
Whole Grains, ≥0.41 oz	0.0048				0.0156			
Sodium, ≤2.17 g	0.0005				0.0019			
Empty Calories, ≤88.87 calorie	0.0004				<0.0001			
**3 HEI Factors (Final Model)**								
(Intercept)	0.0109	0.17	34.16	0.90	0.0168	0.17	34.16	0.90
Whole Grains, ≥0.41 oz	0.0042				0.0058			
Sodium, ≤2.17 g	<0.0001				<0.0001			
Empty Calories, ≤88.87 calorie	0.0013				0.0011			
**2 Diet Factors**								
(Intercept)	0.8901	0.27	36.68	0.73	<0.0001	0.27	36.68	0.73
High-school Diets	0.9332				<0.0001			
Smoothie Diets	0.8931				<0.0001			
**3 HEI and 1 Diet Factors**								
(Intercept)	0.8918	0.17	35.29	0.91	<0.0001	0.17	35.30	0.91
Whole Grains, ≥0.41 oz	0.0108				0.0164			
Sodium, ≤2.17 g	0.0002				0.0003			
Empty Calories, ≤88.87 calorie	0.0007				0.0012			
Smoothie Diets	0.8745				<0.0001			

Note. MR: Misclassification rate; AICc: Akaike's information criterion with corrections; AUC: Area under the curve; HEI: Healthy Eating Index.

Supplementary Table S6. Progression on selecting significant factors contributing to standardized carbohydrates per median number of meals (≤33.95 g) needed for glycemic load (GL) < 20.

Parameters, median units	Logistic Regression with Validation				Generalized Regression Elastic Net Validation			
	p (χ^2)	MR	AICc	AUC	p (χ^2)	MR	AICc	AUC
**Carbohydrate / number of meals needed for GL <20, ≤33.95 (median)**								
**2 HEI Factors**								
(Intercept)	0.0005	0.33	39.91	0.78	0.0007	0.33	39.92	0.78
Total Fruits, ≥0.43 cup	0.0045				0.0045			
Whole Grains, ≥0.41 oz	0.0002				0.0003			
**3 Diet Factors (Final Model)**								
(Intercept)	0.8374	0.20	35.09	0.81	<0.0001	0.20	35.09	0.81
Canned Food Diets	0.0082				0.0082			
Mexican Diets	0.8907				<0.0001			
Smoothie Diets	0.0004				0.0004			
**2 HEI and 1 Diet Factors**								
(Intercept)	0.8849	0.30	36.33	0.88	<0.0001	0.30	36.33	0.88
Total Fruits, ≥0.43 cup	0.0009				0.0009			
Whole Grains, ≥0.41 oz	0.0039				0.0037			
Mexican Diets	0.8977				<0.0001			

Note. MR: Misclassification rate; AICc: Akaike's information criterion with corrections; AUC: Area under the curve; HEI: Healthy Eating Index.

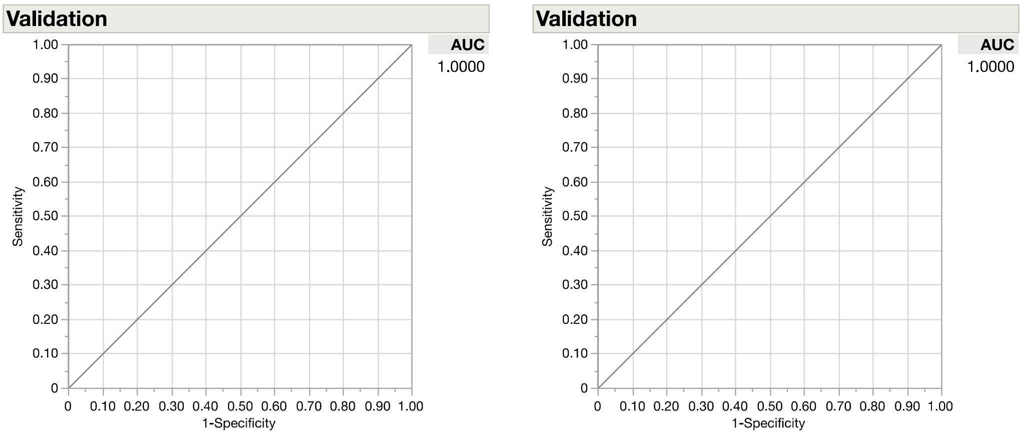

(**a**) Logistic Regression with Validation (**b**) Elastic Net with AICc Validation

Figure S1. Predictors of Healthy Eating Index (80), including 3 HEI factors (whole fruit, whole grains, and empty calories): Area under the receiver operating characteristic curve (AUC) for baseline (a) logistic regression model; (b) Elastic Net with Akaike's information criteria with correction (AICc) validation model.

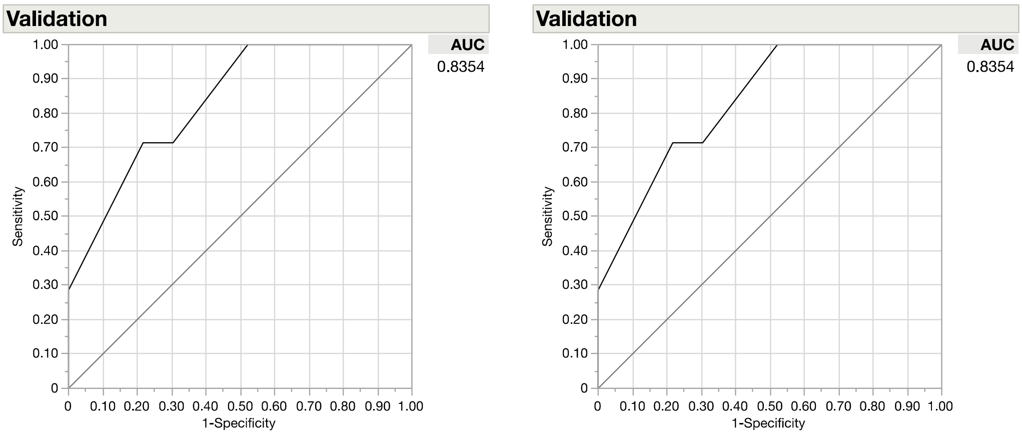

(a) Logistic Regression with Validation (**b**) Elastic Net with AICc Validation

Figure S2. Predictors of Glycemic Index (55), including 1 HEI (total fruit), 1 Caloric (carbohydrate), and 1 Diet Factors (Mexican diet): Area under the receiver operating characteristic curve (AUC) for baseline (a) logistic regression model; (b) Elastic Net with Akaike's information criteria with correction (AICc) validation model.

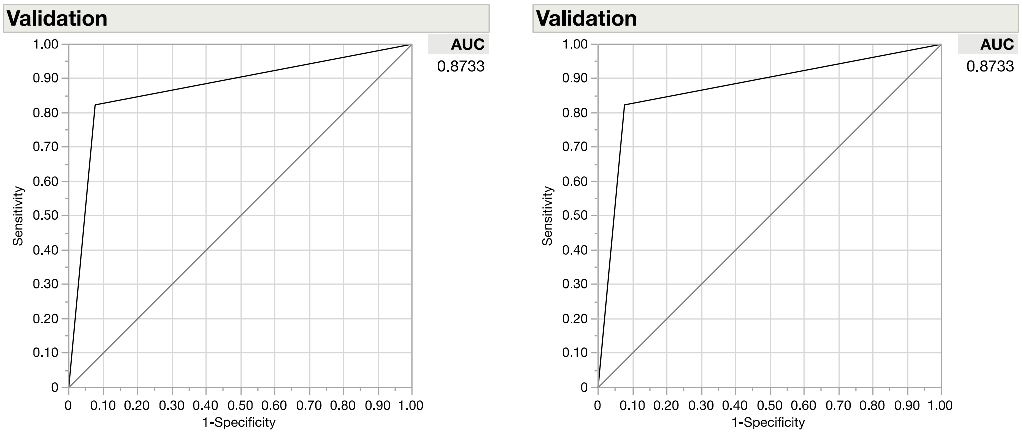

(**a**) Logistic Regression with Validation (**b**) Elastic Net with AICc Validation

Figure S3. Predictors of Glycemic Load (71.8), Carbohydrate: Area under the receiver operating characteristic curve (AUC) for baseline (a) logistic regression model; (b) Elastic Net with Akaike's information criteria with correction (AICc) validation model.

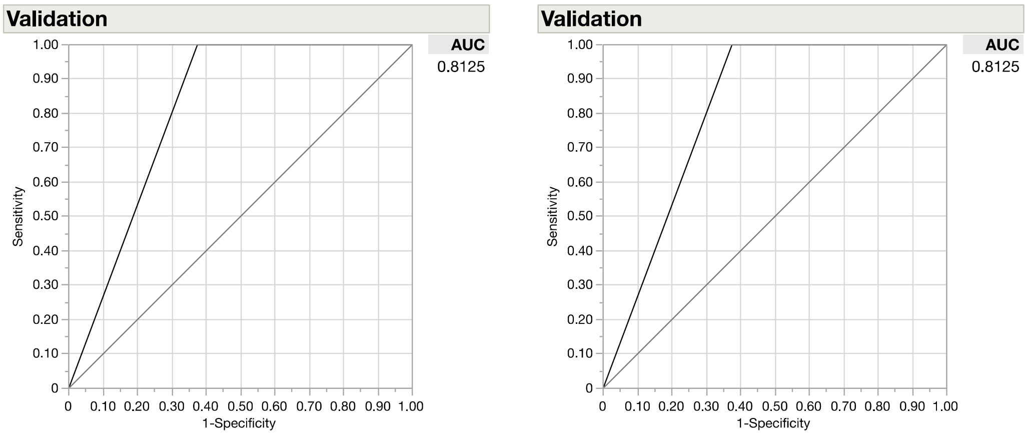

(**a**) Logistic Regression with Validation (**b**) Elastic Net with AICc Validation

Figure S4. Predictors of standardized carbohydrates per number of meals needed for GL <20, (≤33.95 g), including 3 Diet Factors (canned food, Mexican, and smoothie diets): Area under the receiver operating characteristic curve (AUC) for baseline (a) logistic regression model; (b) Elastic Net with Akaike's information criteria with correction (AICc) validation model.
